# Electronic structure and topological properties of centrosymmetric MoAs_2_/WAs_2_ from first principles

**DOI:** 10.1038/s41598-017-10939-1

**Published:** 2017-09-05

**Authors:** Jia Chen, Yu-Ke Li, Jianhui Dai, Chao Cao

**Affiliations:** Condensed Matter Group, Department of Physics, Hangzhou Normal University, Hangzhou, 310036 P. R. China

## Abstract

We investigate the electronic structure of group VI-B transition metal di-arsenides (TAs_2_, T = Mo, W). By comparing the formation energies, the centrosymmetric di-arsenides compounds are energetically more stable, in contrast to the di-phosphorides (MoP_2_/WP_2_). Both compounds can be well described by a two-band model with a pair of well-separated electron/hole bands. The electron/hole carrier density is nearly compensated in MoAs2 (|*n*_*e*_ − *n*_*h*_|/*n*_*h*_ < 1%). The ${{\mathscr{Z}}}_{2}$ classification for all partially occupied bands are topologically strong (1;001), and therefore robust surface states are expected in these materials. Using the adaptive K-mesh method, no energy degenerate state could be found except the spin degeneracy in the whole Brillouin zone, excluding the possibility of intrinsic Dirac or Weyl points near the Fermi level in the system.

## Introduction

The development of topological band theory^1–3^ has introduced a new dimension for electronic structure study in condensed matter physics. Protected by certain symmetries, robust surface states with properly defined chirality emerge at the boundaries separating materials with different topological properties, exemplified by topological insulators^4, 5^. For topological insulators, the bulk states are fully gapped while the surface states are insensitive to local impurities, giving rise to resistivity plateau at low temperatures.

More recently, the study of the topological semimetals^6^, including the Dirac semimetals and the Weyl semimetals, has attracted much attention. Graphene could be regarded as typical example of the Dirac semimetals (DSM), whose band structure are fully gapped except a 4-fold degenerate state at the Fermi level and *K* (i.e., the Dirac point). Close to the Dirac point, the energy dispersion is linear, and the quasi-particle excitations can be well described by the Dirac equations, hence the name. If (in 3-dimensional or 1-dimensional system) the 4-fold degeneracy is lifted and separates into a pair of 2-fold degeneracy with opposite well defined chiralities (Weyl points), either by breaking time-reversal or inversion symmetry, it becomes the so-called Weyl semimetal (WSM). Minimal models as well as the stability of WSMs have also been investigated^7, 8^. Due to the linear energy dispersions, both the DSMs and the WSMs will exhibit ultrahigh mobility and large unsaturated positive magnetoresistivity (MR) with linear dependence on the external magnetic field^9^. In addition, the presence of Weyl fermions in WSMs will lead to angle-dependent negative MR when the current direction is parallel to the external magnetic field due to the Adler-Bell-Jackiw anomaly or “chiral anomaly”^10–13^. Although the WSMs are originally proposed in centrosymmetric magnetic pyrochlore iridates compound^14^, it is now realized in noncentrosymmetric nonmagnetic TaAs compounds^15^, WTe_2_ compounds^16^, MoP_2_ compounds^17^, as well as noncentrosymmetric magnetic GdPtBi^18^.

**Figure 1:**
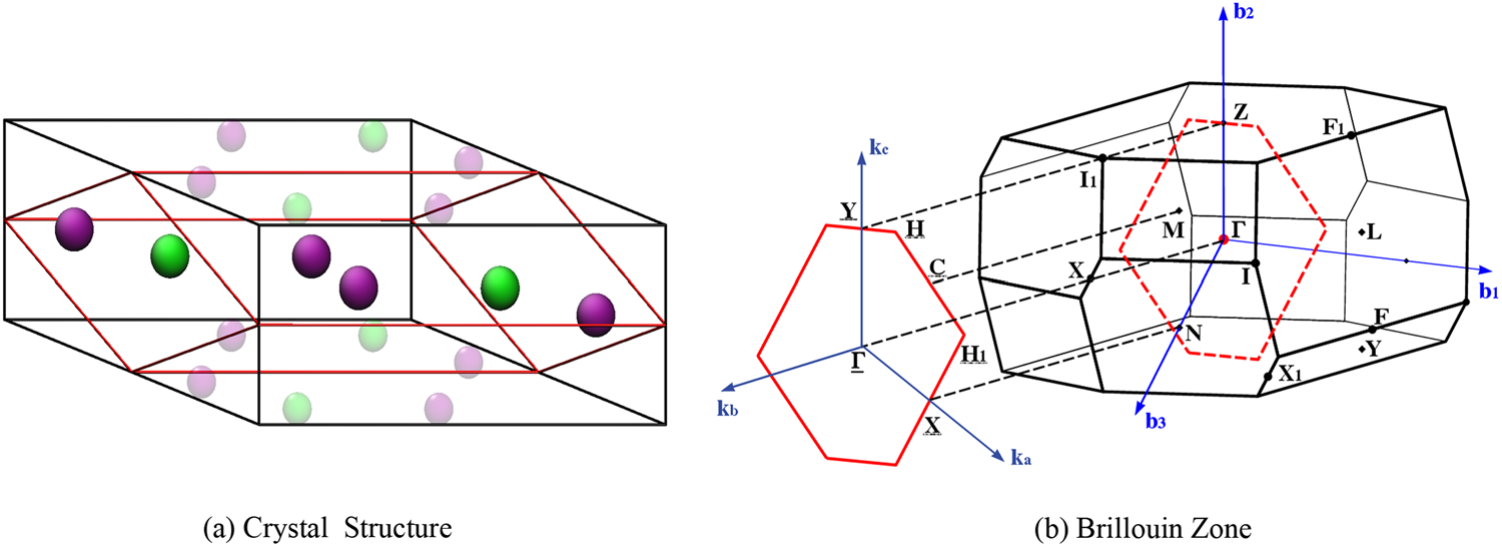
(**a**) Crystal structure of *T* As_2_ (*T* = Mo, W). Yellow atoms are Mo/W, and purple atoms indicate As. The black lines indicate conventional cell while the red lines are primitive cell. The transparent atoms do not belong to the primitive cell. (**b**) Primitive Brillouin zone (BZ), the high symmetry points and choice of high symmetry lines in band structure calculations. The red solid lines indicate the two-dimensional BZ of $[010]$ surface.

However, recent experiments have also observed angular dependent negative MR in non-magnetic, centrosymmetric materials, including Na_3_Bi^19^, Cd_3_As_2_^20, 21^, ZrTe_5_^22^. It was proposed that the effect of external field can also break the time reversal symmetry, and in certain cases leads to the separation of a Dirac point into a pair of Weyl points, while in other cases leads to the formation of nodal rings. The appearance and position of Weyl points in these materials depends on the external field properties (direction, magnitude, etc)^23^, and is therefore distinct from the “intrinsic” WSMs. Similarly, recent experiments have also reported angular dependent negative MR in MoAs_2_ compounds as well as resistivity plateau at low temperatures and nonsaturating postivie MR^24^. Unlike the predicted intrinsic WSM MoP_2_, MoAs_2_ crystallizes in a centrosymmetric structure identical to TaSb_2_ (Fig. 1(a)), another compound exhibiting similar transport properties^25^. Thus, it is probable that they could also be regarded as one of the “extrinsic” WSMs as described earlier. Given the existence of both noncentrosymmetric MoP_2_ and centrosymmetric MoAs_2_, a detailed band structure analysis is necessary.

In this article, we report our latest first-principles calculation results of MoAs_2_ and WAs_2_. The calculation shows that the electron/hole carrier densities are nearly compensated in MoAs_2_, which can serve as natural explanation for the large positive MR. We show that the ${{\mathscr{Z}}}_{2}$ classification for these compounds are strongly nontrivial (1;001), and that no accidental degeneracy (Dirac or Weyl point) can be found in the whole Brillouin zone without external magnetic field.

## Results and Discussion

MoP_2_/WP_2_ were investigated previously by Autès *et al*.^17^. The phosphorus compounds are intrinsic WSMs hosting robust Weyl points due to their non-centrosymmetric crystal structure. In contrast, the arsenic compounds *T* As_2_ (*T* = Mo, W) crystallize in the same structure as TaSb_2_ (Fig. 1(a)), which is base-centered monoclinic with centrosymmetric space group *C*2/*m* (#12) (Table 1). Therefore, they cannot host Weyl points since both the time reversal symmetry and inversion symmetry are preserved in these compounds. We adopt the same bulk and surface Brillouine zone as well as the high symmetry points as defined in ref. 26. After full structure optimization, the lattice constants and internal atomic positions from calculation can be well compared with experimental values within 5% errorbar (Table 1). To further investigate the possible crystalline structures of these pnictides, we have also performed calculations for *T* P_2_ with hypothetical centrosymmetric crystal structure (space group *C*2/*m*). The centrosymmetric MoP_2_ (WP_2_) is energetically ~80 meV/f.u. (30 meV/f.u.) higher than the actual non-centrosymmetric MoP_2_ (WP_2_). Similarly, the noncentrosymmetric MoAs_2_ (WAs_2_) is energetically ~120 meV/f.u. (205 meV/f.u.) higher than centrosymmetric MoAs_2_ (WAs_2_). Therefore, the noncentrosymmetric MoAs_2_/WAs_2_ and centrosymmetric MoP_2_/WP_2_ are energetically unstable at ambient pressure, consistent with the experimental observation.

**Table 1:** Optimized geometry parameters of *T* As_2_ compounds.

	MoAs_2_ (expt)	MoAs_2_ (calc)	WAs_2_ (expt)	WAs_2_ (calc)
*a* (Å)	9.064	9.153	9.079	9.178
*b* (Å)	3.295	3.326	3.318	3.347
*c* (Å)	7.715	7.760	7.692	7.734
*β*	119.37	119.14	119.43	119.24
atom coordinates				
*T* (4i)	(0.154, 0, 0.200)	(0.1529, 0, 0.2010)	(0.154, 0, 0.200)	(0.1517, 0, 0.1999)
As^I^ (4i)	(0.143, 0, 0.531)	(0.1467, 0, 0.5328)	(0.143, 0, 0.531)	(0.1462, 0, 0.5331)
As^II^ (4i)	(0.399, 0, 0.112)	(0.4026, 0, 0.1065)	(0.399, 0, 0.112)	(0.4031, 0, 0.1083)

*β* is the angle formed by **a** and **c** lattice vectors. Columns with “(calc)” lists results from calculation, while columns with “(expt)” are experimental values from refs 38 and 39, for comparison. The lower part of the table lists the atomic coordinates. *T* indicates the transition metal (Mo or W); (4i) after the element name indicates the Wyckoff letters of the atomic site.

### Band Structure and Density of States

**Figure 2:**
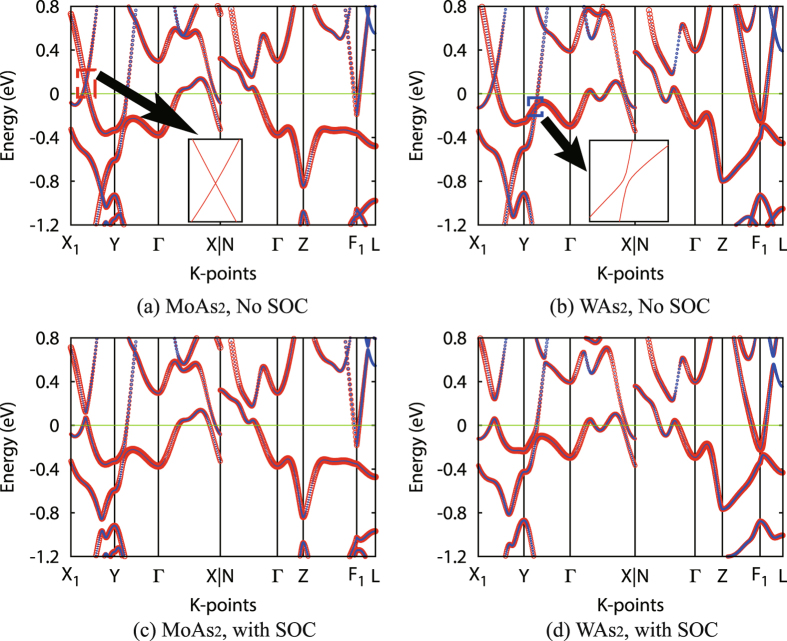
Band structure of (**a**,**c**) MoAs_2_ and (**b**,**d**) WAs_2_ calculated without SOC (**a**,**b**) and with SOC (**c**,**d**). The widths of lines are proportional to orbital contributions; red and blue color indicate Mo-4d/W-5d and As-4p contributions, respectively. Insets are details obtained by calculating 1000 K-points from X_1_ to Y (Y to Γ) in panel (a) (panel (b)), respectively.

Before performing further analysis, it would be instructive to compare the band structure of *T* As_2_ compounds calculated with/without spin-orbit coupling (SOC). Since Mo and W belong to the same family, the band structure of both compounds are very close to each other. Dirac-like dispersion features can be identified between X_1_ and Y from both *T* As_2_ band structure without SOC (red dashed box in Fig. 2a). The specific feature is ~0.1 eV above *E*_*F*_ in MoAs_2_ (~0.2 eV above *E*_*F*_ in WAs_2_). Please be noted that the feature (blue dashed box in Fig. 2b) at ~0.1 eV below *E*_*F*_ in WAs_2_ is not band-crossing feature (inset of Fig. 2(b)), although the two bands are extremely close to each other. More band-crossing features could be identified at much lower energies (below *E*_*F*_-0.8 eV), between X_1_ and Y as well as F_1_ and L, but they are far from the Fermi level and therefore may not be relevant with transport properties. Close to the Fermi level, the electronic states of both compounds are dominated by Mo-4d or W-5d orbitals (red color in Fig. 2), with moderate As-4p contributions (blue color in Fig. 2). The band structure is then fitted to a tight-binding Hamiltonian using the maximally localized Wannier function (MLWF) method. Using the adaptive K-mesh searching method, it is then determined that the band-crossings close to *E*_*F*_ form nodal rings in the BZ.

All above mentioned band-crossing features are fully gapped out when SOC is considered in the calculation. For the MoAs_2_ compound, since neither Mo nor As is heavy element, the SOC splitting is fairly small (~50 meV for the feature between X_1_ and Y); while for the WAs_2_ compound, the SOC splitting is much larger (~180 meV for the same feature). The SOC does not significantly alter the orbital component for either compound, thus the electronic states near *E*_*F*_ are still dominated by Mo-4d/W-5d and As-4p orbitals. Using the adaptive K-mesh method, we searched the whole BZ and ruled out the possibility of accidental energy degeneracies in these systems. The electron/hole bands crossing the Fermi level are therefore well separated from each other, as well as from all other bands. Thus, the electron/hole type carrier densities can be evaluated by calculating the electron/hole band contributions to the density of states (DOS). It is therefore estimated that the *n*_*e*_ = 1.90 × 10^21^ cm^−3^ and *n*_*h*_ = 1.92 × 10^21^ cm^−3^ in MoAs_2_ (*n*_*e*_ = 1.85 × 10^21^ cm^−3^ and *n*_*h*_ = 1.72 × 10^21^ cm^−3^ in WAs_2_), where *n*_*e*_ and *n*_*h*_ represents the carrier density of electrons and holes, respectively. Therefore, the deviation from electron-hole compensation is ~1% in MoAs_2_ with slightly more holes, whereas the deviation is ~7.4% in WAs_2_ with slightly more electrons. From the two-band model with both electron and hole contributions, the Hall coefficient at small field limit and the magnetoresistivity (MR)^27^ reads

1 $${R}_{H}=\frac{1}{e}\frac{{\mu }_{h}^{2}{n}_{h}-{\mu }_{e}^{2}{n}_{e}}{{({\mu }_{e}{n}_{h}+{\mu }_{h}{n}_{e})}^{2}}$$

and

2 $$MR=\frac{{n}_{e}{n}_{h}{\mu }_{e}{\mu }_{h}{({\mu }_{e}+{\mu }_{h})}^{2}{B}^{2}}{{({n}_{e}{\mu }_{e}+{n}_{h}{\mu }_{h})}^{2}+{({n}_{e}-{n}_{h})}^{2}\,{({\mu }_{e}{\mu }_{h})}^{2}{B}^{2}}$$

where *μ*_*e*_ and *μ*_*h*_ are the mobilities of electron-type and hole-type carriers, respectively. Therefore, a nearly compensated metal with slightly more hole-type carrier can also exhibit negative *R*_*H*_ if electron mobility is sufficiently larger than hole mobility (*μ*_*e*_/*μ*_*h*_ > 1.005 for MoAs_2_), as well as *B* ^2^-dependent MR at small field. If the deviation from electron-hole compensation is very small, the magnetic field required to saturate MR can be very large. These arguments are in consistency with the experimental observations of MoAs_2_, and thus we believe that the system can be described by a two-band model with both electron and hole contributions.

### Topological Invariants and Surface States

**Figure 3:**
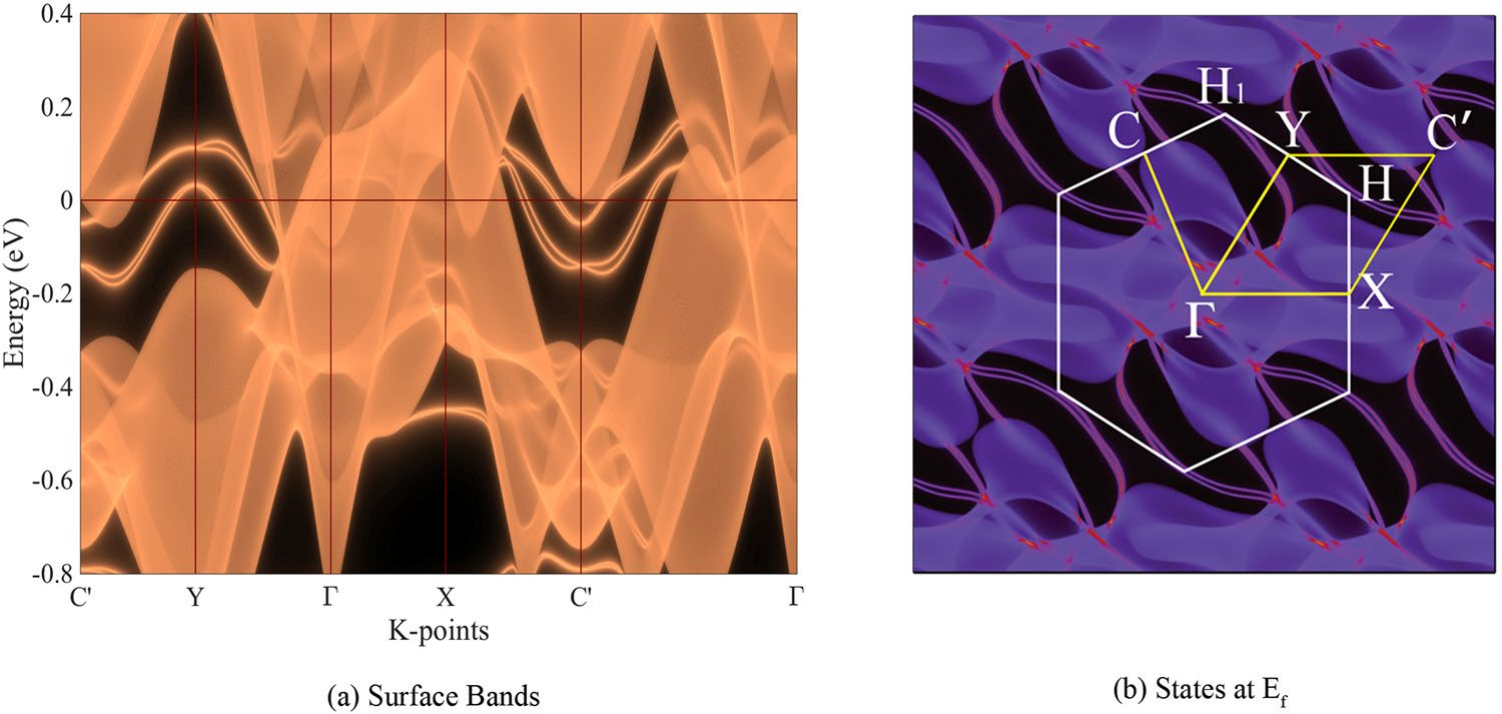
Electronic states of MoAs_2_ at[010] surface. The white lines in panel (b) encloses the first BZ of[010] surface, with definitions of its high symmetry points (C’ is equivalent to C). (**a**) Surface band structure obtained using the Green’s function method, along the high symmetry line indicated by the yellow lines in panel (b). The Fermi level is aligned at 0 eV. (**b**) Surface states at *E*_*F*_.

Since the SOC gapped out all nodal points between the electron and hole bands, and separated them from all other bands, the topological invariant ${{\mathscr{Z}}}_{2}$ can be well defined for the system. As the system has an inversion center, its ${{\mathscr{Z}}}_{2}$ invariant can be determined by calculating the parities of band states at 8 time-reversal invariant momenta (TRIM)^28^ (Table 2). The resulting ${{\mathscr{Z}}}_{2}$ classification counting up to the partially filled hole (band 12)/electron band (band 13) is (0;000) and (1;001), respectively. Therefore the band structure of both MoAs_2_ and WAs_2_ are topologically strong, and robust surface states between band 13 and 14 immune to impurities are expected. However, since both MoAs_2_ and WAs_2_ are indeed very metallic systems, they are not topological insulators, and the appearance of these surface states are not guaranteed, similar to the case in TaSb_2_^26^. Using the surface green’s function method, we calculated the electronic states of MoAs_2_ at $[010]$ surfaces (Fig. 3). From Fig. 1b, it is clear that the bulk states from Γ, X, Y and X_1_ will be projected to the 2D BZ center $\underline{{\rm{\Gamma }}}$; bulk states from Z, L, I and I_1_ will be projected to $\underline{{\rm{Y}}}$; M to $\underline{{\rm{C}}}$; N to $\underline{{\rm{X}}}$. The surface states are completely overwhelmed by the bulk states along Γ to $\underline{{\rm{X}}}$, but they could be observed around $\underline{{\rm{Y}}}$, $\underline{{\rm{C}}}$ and $\underline{{\rm{H}}}$, respectively. Unfortunately, all the surface states crossing the Fermi levels are not topological, since none of them connects between bulk band 13 and 14.

**Table 2:** Band parities at time-reversal invariant momenta (TRIM).

	*ξ*_*i*_ for MoAs_2_			Π_12_	Π_13_	*ξ*_*i*_ for WAs_2_			Π_12_	Π_13_
Γ	+ − + + −	+ − + + +	− − − −	−	+	+ − + + −	+ − + + +	− − − −	−	+
N	− + + − −	− + + − +	+ + − +	−	+	+ − + − −	− + + − +	+ + − +	−	+
Y	− + − + −	− + + − +	+ + − −	−	+	− + − + −	− + + − +	+ + − −	−	+
Z	− + + − −	− + + + −	− + − −	+	−	− + + − −	− + + + −	− + − −	+	−
M	− + + − −	+ − − + +	− + + −	+	+	+ − − + −	+ − − + +	− + + −	+	+
L	− + − + +	− + − + −	+ − + −	+	+	− + − + +	− + − + −	+ − + −	+	+

The labels for TRIMs are: Γ (0, 0, 0); N (*π*, 0, 0) and (0, *π*, 0); Y (*π*, *π*, 0); Z (0, 0, *π*); M (*π*, 0, *π*) and (0, *π*, *π*); and L (*π*, *π*, *π*). *ξ*_*i*_ are parity eigenvalues for each Kramer pair in increasing energy order. Only valence band states (*ε*_*ik*_ > *E*_*F*_-8.0 eV) are listed. ${{\rm{\Pi }}}_{n}={{\rm{\Pi }}}_{i=1}^{n}\,{\xi }_{i}$ can be used to determine the ${{\mathscr{Z}}}_{2}$ topological invariant, and the hole (electron) band crossing *E*_*F*_ is *n* = 12 (13). Π_12_ and Π_13_ are exactly the same for both compounds.

Again, MoAs_2_ and WAs_2_ are isostructural to another family of compounds represented by TaSb_2_, where both large positive MR and longitudinal negative MR have been also observed^25, 29^ although the origin is still under debate^30–33^. In fact, the band structure of NbAs_2_ is similar to the MoAs_2_ band structure with *E*_*F*_ shifted 1 eV lower. The shift of the Fermi level is equivalent to losing 2 electrons per unit cell, or 1 electron per formula unit in the rigid band approximation. Such similarities can be found between WAs_2_ and TaAs_2_ likewise. Moreover, the polarization Π_*n*_ for 13 ≥ *n* ≥ 11 at TRIM for NbAs_2_ or TaAs_2_ are exactly the same as MoAs_2_ or WAs_2_. Thus, MoAs_2_ (WAs_2_) can be regarded as electron-doped NbAs_2_ (TaAs_2_). Previous theoretical study has demonstrated that Weyl points can be induced by external magnetic field in TaAs_2_ system^23^. Such phenomena is also possible in MoAs_2_ or WAs_2_, leading to longitudinal negative MR observed in experiments. In addition, since the ${{\mathscr{Z}}}_{2}$ invariant up to band 13 is strongly topological, topological insulator state is possible in these structures, if the system could be electron doped to band 13 while maintaining the direct gap between band 13 and 14.

## Conclusion

Using the first-principles calculation method, we have studied the electronic structure and topological properties of MoAs_2_ and WAs_2_. Despite of their similar chemical compositions to MoP_2_, these arsenides are centrosymmetric, and thus cannot hold intrinsic Weyl points. Both compounds could be regarded as two band system with both electron and hole carriers. The carriers are nearly compensated in MoAs_2_, which may lead to large positive MR proportional to *B* ^2^. The ${{\mathscr{Z}}}_{2}$ classification of these materials is (1;001), and robust surface states are expected. Additional degenerate states other than spin degeneracy are absent throughout the whole Brillouin zone, meaning the system do not hold intrinsic Dirac nor Weyl points.

## Methods

The electronic structure of these compounds were calculated using density functional theory employing plane-wave basis projected augmented wave (PAW) method as implemented in Vienna Abinitio Simulation Package (VASP)^34, 35^. After careful convergence tests, we found that an energy cut-off of 400 eV and a 16 × 16 × 7 Γ-centered K-mesh (over primitive cell) is sufficient to converge the total energy to 1 meV/atom. The electronic structure calculations were then performed using crystal structures with optimized lattice constants and internal atomic parameters. The density of states (DOS) were obtained with dense Γ-centered K-mesh of 32 × 32 × 14 and tetrahedron method. With the maximally localized wannier function (MLWF) method^36^, the DFT band structure is extrapolated to a 100 × 100 × 100 K-mesh to construct the Fermi surface. The resulting tight-binding Hamiltonian was also used to calculate the surface states using the surface Green’s function^37^. ${{\mathscr{Z}}}_{2}$ topological indices were calculated using the parity-check method^28^.

The band anti-crossing features, or energy degeneracy points (nodal points) in the current study, are identified using an adaptive K-mesh searching method. It begins with interpolating band energies on a dense 100 × 100 × 100 K grid using a well fitted tight-binding Hamiltonian (with MLWF method in the current study). Then the vicinity of the K-points yielding nearly degenerate eigen-energies ($\delta \varepsilon =|{\varepsilon }_{k}^{n+1}-{\varepsilon }_{k}^{n}| < 0.01\,{\rm{eV}}$) will be extrapolated to a 1000 times denser K grid (10 times in each direction) and the K-point yielding smallest *δε* will be kept as the initial guess of the next iteration. After 3 iterations, the K-points yielding *δε* < 1.0 × 10^−5^ eV will be regarded as potential candidates for nodal points. Eventually, all candidates are cross-checked with DFT calculations to verify the energy differences.

## References

[1] Hasan MZ, Kane CL (2010). *Colloquium*: Topological insulators. Rev. Mod. Phys..

[2] Qi X-L, Zhang S-C (2011). Topological insulators and superconductors. Rev. Mod. Phys..

[3] Bansil A, Lin H, Das T (2016). *Colloquium*: Topological band theory. Rev. Mod. Phys..

[4] Zhang H (2009). Topological insulators in bi2se3, bi2te3 and sb2te3 with a single dirac cone on the surface. Nat Phys.

[5] Xia Y (2009). Observation of a large-gap topological-insulator class with a single dirac cone on the surface. Nat Phys.

[6] Burkov AA (2016). Topological semimetals. Nat Mater.

[7] Morimoto T, Furusaki A (2014). Weyl and dirac semimetals with *F*_2_ topological charge. Phys. Rev. B.

[8] McCormick TM, Kimchi I, Trivedi N (2017). Minimal models for topological weyl semimetals. Phys. Rev. B.

[9] Liang T (2015). Ultrahigh mobility and giant magnetoresistance in the dirac semimetal cd3as2. Nat Mater.

[10] Adler SL (1969). Axial-vector vertex in spinor electrodynamics. Phys. Rev..

[11] Bell JS, Jackiw R (1969). A pcac puzzle: *π*0 → *γγ* in the *σ*-model. Il Nuovo Cimento A (1965–1970).

[12] Son DT, Spivak BZ (2013). Chiral anomaly and classical negative magnetoresistance of weyl metals. Phys. Rev. B.

[13] Burkov AA (2015). Chiral anomaly and transport in weyl metals. Journal of Physics: Condensed Matter.

[14] Wan X, Turner AM, Vishwanath A, Savrasov SY (2011). Topological semimetal and fermi-arc surface states in the electronic structure of pyrochlore iridates. Phys. Rev. B.

[15] Weng H, Fang C, Fang Z, Bernevig BA, Dai X (2015). Weyl semimetal phase in noncentrosymmetric transition-metal monophosphides. Phys. Rev. X.

[16] Soluyanov AA (2015). Type-ii weyl semimetals. Nature.

[17] Autès G, Gresch D, Troyer M, Soluyanov AA, Yazyev OV (2016). Robust type-ii weyl semimetal phase in transition metal diphosphides *x*p_2_ (*x* = Mo, w). Phys. Rev. Lett..

[18] Hirschberger M (2016). The chiral anomaly and thermopower of weyl fermions in the half-heusler gdptbi. Nat Mater.

[19] Xiong J (2015). Evidence for the chiral anomaly in the dirac semimetal na3bi. Science.

[20] Li, H. *et al*. Negative magnetoresistance in dirac semimetal cd3as2. *Nature Communications* **7**, 10301 EP– (2016). 10.1038/ncomms10301 PMC4729874 26744088

[21] Li, C.-Z. *et al*. Giant negative magnetoresistance induced by the chiral anomaly in individual cd3as2 nanowires. *Nature Communications* **6**, 10137 EP– (2015). 10.1038/ncomms10137 PMC4703844 26673625

[22] Li Q (2016). Chiral magnetic effect in zrte5. Nat Phys.

[23] Gresch, D., Wu, Q., Winkler, G. & Soluyanov, A. Hidden weyl points in centrosymmetric paramagnetic metals. *New Journal of Physics* (2017).

[24] Wang, J. *et al*. Magnetoresistance and robust resistivity plateau in MoAs2. *ArXiv e*-*prints* arXiv:1610.08594 (2016). 10.1038/s41598-017-15962-w PMC5688174 29142314

[25] Li Y (2016). Resistivity plateau and negative magnetoresistance in the topological semimetal tasb_2_. Phys. Rev. B.

[26] Xu C (2016). Electronic structures of transition metal dipnictides *xpn*_2_ (*x* = Ta, nb; *pn* = P, as, sb). Phys. Rev. B.

[27] Pippard, A. B. *Magnetoresistance in Metals*, pp 29 (Cambridge University Press, 1989).

[28] Fu L, Kane CL (2007). Topological insulators with inversion symmetry. Phys. Rev. B.

[29] Shen B, Deng X, Kotliar G, Ni N (2016). Fermi surface topology and negative longitudinal magnetoresistance observed in the semimetal nbas_2_. Phys. Rev. B.

[30] Wang, Y.-Y., Yu, Q.-H. & Xia, T.-L. Resistivity plateau and extremely large magnetoresistance in NbAs2 and TaAs2. *ArXiv e*-*prints* arXiv:1601.04239 (2016).

[31] Luo, Y. *et al*. Anomalous electronic structure and magnetoresistance in taas2. *Scientific Reports* **6**, 27294 EP– (2016). 10.1038/srep27294 PMC4895157 27271852

[32] Yuan Z, Lu H, Liu Y, Wang J, Jia S (2016). Large magnetoresistance in compensated semimetals taas_2_ and nbas_2_. Phys. Rev. B.

[33] Li, Y. *et al*. Negative Magnetoresistance in Topological Semimetals of Transition-Metal Dipnictides with Nontrivial Z2 Indices. *ArXiv e*-*prints* arXiv:1603.04056 (2016).

[34] Kresse G, Hafner J (1993). *Ab initio* molecular dynamics for liquid metals. Phys. Rev. B.

[35] Kresse G, Joubert D (1999). From ultrasoft pseudopotentials to the projector augmented-wave method. Phys. Rev. B.

[36] Souza I, Marzari N, Vanderbilt D (2001). Maximally localized wannier functions for entangled energy bands. Phys. Rev. B.

[37] Sancho MPL, Sancho JML, Sancho JML, Rubio J (1985). Highly convergent schemes for the calculation of bulk and surface green functions. Journal of Physics F: Metal Physics.

[38] Moas_2_ crystal structure: Datasheet from “pauling file multinaries edition – 2012” in springermaterials http://materials.springer.com/isp/crystallographic/docs/sd_0530117.

[39] Was_2_ crystal structure: Datasheet from “pauling file multinaries edition – 2012” in springermaterials http://materials.springer.com/isp/crystallographic/docs/sd_0529062.

